# Line-Field Confocal Optical Coherence Tomography Evaluation of Eyelid Skin Lesions

**DOI:** 10.3390/diagnostics13233590

**Published:** 2023-12-03

**Authors:** Alessandro Di Stefani, Simone Cappilli, Giovanni Cuffaro, Bruno Fionda, Monica Maria Pagliara, Andrea Paradisi, Costantino Ricci, Ernesto Rossi, Maria Grazia Sammarco, Giovanni Schinzari, Luca Tagliaferri, Maria Antonietta Blasi, Elisa Cinotti, Alessandro Moro, Gustavo Savino, Mariano Suppa, Ketty Peris

**Affiliations:** 1 UOC di Dermatologia, Dipartimento di Scienze Mediche e Chirurgiche, Fondazione Policlinico Universitario A. Gemelli IRCCS, 00168 Rome, Italy; alessandro.distefani@gmail.com (A.D.S.); aparad78@gmail.com (A.P.); ketty.peris@unicatt.it (K.P.); 2 Ocular Oncology Unit, Università Cattolica del Sacro Cuore, 00168 Rome, Italy; 3 Ocular Oncology Unit, Fondazione Policlinico Universitario A. Gemelli IRCCS, 00168 Rome, Italy; giovanni.cuffaro@guest.policlinicogemelli.it (G.C.); monicamaria.pagliara@policlinicogemelli.it (M.M.P.); mariagrazia.sam@gmail.com (M.G.S.); mariaantonietta.blasi@policlinicogemelli.it (M.A.B.); gustavo.savino@unicatt.it (G.S.); 4 Ophthalmology Unit, Università Cattolica del Sacro Cuore, 00168 Rome, Italy; giovanni.schinzari@policlinicogemelli.it (G.S.); alessandro.moro@policlinicogemelli.it (A.M.); 5 UOC Radioterapia Oncologica, Dipartimento di Diagnostica per Immagini, Radioterapia Oncologica ed Ematologia, Fondazione Policlinico Universitario A. Gemelli IRCCS, 00168 Rome, Italyluca.tagliaferri@policlinicogemelli.it (L.T.); 6 Pathology Unit, Maggiore Hospital, AUSL Bologna, 40133 Bologna, Italy; costanricci@gmail.com; 7 Department of Experimental, Diagnostic and Specialty Medicine (DIMES), S. Orsola-Malpighi University Hospital, University of Bologna, 40138 Bologna, Italy; 8 Medical Oncology Unit, Fondazione Policlinico Universitario A. Gemelli IRCCS, 00168 Rome, Italy; ernestorossi.rm@gmail.com; 9 Dermatology Unit, Department of Medical, Surgical and Neurological Sciences, Università degli Studi di Siena, 53100 Siena, Italy; elisacinotti@gmail.com; 10 Groupe d’Imagerie Cutanée Non Invasive (GICNI) of the Société Française de Dermatologie (SFD), 75008 Paris, France; dr.marianosuppa@gmail.com; 11 Maxillo-Facial Surgery Unit, Fondazione Policlinico Universitario A. Gemelli IRCCS, 00168 Rome, Italy; 12 Department of Dermatology, Hôpital Erasme, Université Libre de Bruxelles, 1050 Brussels, Belgium; 13 Department of Dermatology, Institut Jules Bordet, Université Libre de Bruxelles, 1050 Brussels, Belgium

**Keywords:** melanoma, histology of the skin, line-field confocal optical coherence tomography, eyelid, epithelial carcinomas, non-invasive diagnosis, skin cancers

## Abstract

Background: Periocular malignancies may be clinically different from the examples arising at other sites, with possible delayed diagnosis and greater challenges for treatment and repair. Line-field confocal optical coherence tomography (LC-OCT) is a recently developed technique characterized by an unprecedented capacity to acquire high-definition images in vertical and horizontal modes. In this study, we aimed to investigate the LC-OCT morphological features of a series of eyelid skin lesions, correlating them to histopathological findings. Methods: Patients with biopsy-proven equivocal skin lesion in the eyelid area, previously investigated by means of LC-OCT, were included in the study. Percentage overall agreement was estimated for LC-OCT and histopathological diagnosis for study cases. Results: A total of 51 patients (28 women, 23 men; mean age 66.4 years old), for a total of 51 skin lesions, were assessed. The histopathological diagnosis consisted of 30 malignant and 21 benign tumors. Different entities were characterized by peculiar findings in LC-OCT, alike to histopathological features, allowing for an accurate “in vivo” classification in almost all cases, with a diagnostic concordance with histopathology of 92.1% (47/51). Conclusions: By integrating this new imaging technique into the assessment of suspicious tumors in this area, diagnostic accuracy may increase, improving strategies adopted in multidisciplinary meetings and patient-centered care.

## 1. Introduction

The eyelid may be affected by a large spectrum of conditions, from benign to malignant tumors, from inflammatory to infectious diseases [1,2]. Although benign lesions are the most common in this area, an unsolved issue remains the differential diagnosis with malignant lesions, and equivocal cases often require an incisional biopsy for a definite diagnosis [3,4,5]. Periocular malignancies may present differently, with more aggressive behavior, posing greater challenges for treatment and repair than cutaneous malignancies at other sites [3,4,5]. Up to 10% of malignant skin cancers developing on the face affect this sensitive area, with basal cell carcinoma (BCC) being the most common neoplasm, followed by squamous cell carcinoma (SCC), cutaneous melanoma (CM), and sebaceous gland carcinoma (SGC) [4]. BCC accounts for 90% of all malignancies and arises with higher rates on the lower eyelid or medial canthus, rather than on the upper eyelid and lateral canthus, with a predominance of nodular and nodulocystic variants in histopathology [6,7].

Eyelid tumors can cause significant morbidity primarily through extensive local invasion, occasionally resulting in orbital and intracranial involvement with eye vision impairment [8]. In this regard, and for an excellent clinical and aesthetic outcome, early recognition is mandatory, followed by traditional or Mohs micrographic surgery where feasible, as the latter has been proven to lower the recurrence rate [9]. However, the treatment of malignancies of the eyelid is an interdisciplinary challenge, and an optimal treatment approach may be facilitated by the discussion between different specialists in multidisciplinary tumor meetings. This approach is likely to be effective in the planning of an integrated therapeutic management, enabling clinicians to discuss imaging results and thus defining a more precise or complete staging by sharing the expertise of different team members [10].

In dermatology, the application of non-invasive diagnostic technologies has been continuously growing; imaging techniques such as dermoscopy, reflectance confocal microscopy (RCM), and conventional optical coherence tomography (OCT) have largely been demonstrated to facilitate the clinical diagnosis of skin malignancies, including tumors occurring in the eyelid area. Indeed, dermoscopy provides further clues with which to better characterize suspicious lesions, also supported, where available, by the in vivo tools RCM and OCT, two complementary systems that reproduce details of skin lesion architecture [7,11,12,13,14]. In recent years, various studies have contributed to supporting the additional value of dermoscopy in the assessment of skin lesions arising in the eyelid or eyelid margins, and although specific features have been reported for certain tumors (i.e., linear vessels arranged perpendicularly to the eyelid margin in BCC), knowledge of dermoscopic patterns in this specific area is limited [11,13]. With regard to handheld RCM, it turned out to be a reliable tool for the in vivo diagnosis of eyelid margin and conjunctival tumors, showing a greater sensitivity and specificity compared to clinical examination alone; moreover, thanks to a small tip enabling the examination of curved surfaces, handheld RCM can be easily repeated at different times during follow-up [14]. However, this technique is not yet available in many referral centres, requires long and dedicated training, and its diagnostic accuracy decreases in inflamed, traumatized, and thick lesions. Indeed, the main limitation of RCM imaging is its relatively low penetration, since this optical device is able to analyze the skin with nuclear and cellular morphology, up to a depth of ~200 μm, which is not sufficient to image the deep dermis. Also problematic is the fact that RCM sections are oriented perpendicular to conventional histological sections, making them difficult to interpret [15]. The OCT device, reproducing images of lesions up to 1 mm in depth within the skin in a vertical plane, overcomes the technical barriers of RCM, historically representing its complementary tool [15]. Its application in the evaluation of suspected tumors in the eyelid area led to the identification of specific patterns that may help to indicate certain diagnoses. In addition, Fourier-domain OCT imaging provided additional information in the recognition of morphological features, further characterizing nodular BCC compared to conventional OCT diagnostic criteria, with a high correlation to histology [7,16].

Recently, a new device, Line-field confocal optical coherence tomography (LC-OCT), was developed and marketed thanks to its extraordinary capacity to acquire high-definition images in vertical and horizontal modes, merging RCM cellular resolution (~1 μm) and OCT depth acquisition (~500 μm) [17]. These unprecedented technical characteristics probably explain why LC-OCT has been gaining momentum in the field of dermatological research, as over the last 5 years, numerous publications have seen the light of the day, including a large spectrum of physiological and pathological dermatological conditions. In particular, in the latter group, this tool has been successfully employed to visualize epidermal and upper dermal alterations of tumoral, inflammatory, autoimmune, vascular, and infective conditions and diseases, and also in a pediatric population [17,18,19,20,21,22,23,24,25,26,27,28]. Skin manifestations of rare genetic disorders have also been assessed [26].

In this study, we aimed to investigate the LC-OCT morphological features of a consecutive series of skin lesions arising on the eyelid area, making a comparison with histopathological slides.

## 2. Materials and Methods

### 2.1. Inclusion Criteria

A consecutive series of adult patients (>18 years old) with equivocal skin lesions in the eyelid area were referred to the dermatology department or the ophthalmology outpatient service of Fondazione Policlinico Universitario A. Gemelli IRCCS, Rome, Italy, from 1 October 2021 to 1 March 2023. Eyelid skin lesions that were surgical excised and previously investigated by means of LC-OCT were retrospectively included in the study. As histopathology represents the gold-standard for a definite diagnosis, all lesions with no pathological report were excluded.

### 2.2. Sample Population

Clinical data were retrospectively obtained based on medical records. Demographic characteristics (age, sex, skin phototype) were recorded as well as tumor characteristics (clinical aspect, anatomical location, lesion size). Clinical and video dermoscopic images (Dermalview Dual, Gavimedica srl, Camposano, Italy) were acquired for each patient before and after surgical excision. This study was carried out in accordance with the Declaration of Helsinki. Ethics committee approval was waived because the study affected neither routine diagnostic nor therapeutic management. The patients in this manuscript have given written informed consent to the publication of their case details. All data were de-identified before use.

### 2.3. LC-OCT Device, Imaging Acquisition, and Evaluation

LC-OCT is an imaging modality based on a combination of low-coherence optical interferometry and reflectance confocal optical microscopy with line illumination and line detection. The CE-marked prototype of LC-OCT (DAMAE Medical, Paris, France) is made of a portable probe connected to a central unit and a display. The acquisition rate is 10 frames/second, with a scanning depth of 500 μm, an axial resolution of 1.2 μm, a lateral resolution of 1.3 μm, and a lateral field of view of 1.2 mm [17]. Live images are directly visualized on the screen while the operator gently moves the tip of the probe over the skin. The device is equipped with a polarized light dermatoscope, providing a field of examination corresponding to a circular surface of 2.5 mm diameter at a 5 µm resolution. A dermoscopic frame is visualized along with the vertical/horizontal LC-OCT image on the device display; the red line and the red rectangle inside the dermoscopic frame, respectively, correspond to the field of view on the vertical and horizontal planes of the LC-OCT laser −1.2 mm examination field and indicate the exact position of the area evaluated when performing the exam. A paper reinforcement ring was used to ensure the correct position of the probe before imaging, and a drop of paraffin oil was applied to the tip of the handheld probe. Paraffin oil provides index matching at the air/glass interface of the glass plate, suppressing the reflection of light.

For each examination, minimal or no pressure was applied on the skin to ensure a correct visualization of the upper layers of the epidermis and of the dermal vessels. Two operators captured vertically and horizontally oriented videos of the study lesions, considering at least four LC-OCT videos (one vertical and three horizontal frames at the epidermis, DEJ, and dermis) as adequate to investigate LC-OCT criteria. Additionally, when available, one 3D video was included in the image evaluation. A 3D cube is derived from a rectangular reconstruction of the skin area (1.2 × 0.5 × 0.5 mm) obtained via software elaboration with MinIP, 3DSlicer, version 4.10.2. The software elaboration also allows us to navigate within the specific area following the 3 axes to assess and measure stain-specific cells/structures in detail [29].

LC-OCT digital acquisitions were carefully reviewed while blind to clinical features, dermoscopy, and histopathology, attempting to identify disease-specific patterns for all cases. Imaging pictures were evaluated by consensus of two investigators experienced in using LC-OCT in their clinical daily routine according to features already described in the literature in different skin conditions [17,19,24,25]. In the case of a discrepancy, the final score for a particular case and structure was obtained based on the decision of a third evaluator. Additionally, previously non-reported LC-OCT findings have been introduced into the analysis based on personal observation. Hence, LC-OCT-based pre-surgical diagnosis was established.

### 2.4. Study Outcome and Statistical Analysis

The primary objective was the description of LC-OCT features in different skin tumors of the eyelid, while the secondary outcome was the concordance of the LC-OCT-based diagnosis with the final histopathologic diagnosis, representing the gold standard.

Descriptive statistics, including means, medians, range, and relative frequencies, were used to describe the study participants and the LC-OCT findings. Percentage overall agreement was estimated for LC-OCT and histologic assessments for the study cases.

## 3. Results

### 3.1. Clinical Features

We included 51 patients (28 women, 23 men; mean age 66.4 years, range 34–88 years) for a total of 51 skin lesions, (Table 1). The patients were of Caucasian origin with I–III Fitzpatrick skin phototype; 32 tumors (63.0%) were diagnosed in patients with I/II skin phototype and 19 (37.0%) in patients with skin phototype III. Malignant lesions correlated with older patients rather than benign lesions (mean 70.0 ± 14.4 years old vs. mean 60.6 ± 15.8 years old; *p* < 0.001). The final histopathological diagnosis comprised 30 malignant and 21 benign skin lesions. Malignant tumors were classified as follows: 24 BBCs, 4 SCCs, and 2 CMs; while benign tumors were classified as follows: 9 seborrheic keratoses (SK), 5 scars, 3 hidrocystomas, 2 actinic keratoses (AK), 1 blue nevus, and 1 cherry hemangioma (CH). On clinical grounds, lesions mainly presented as nodules with ill-defined margins (28/51, 55%). Less common lesions were slightly elevated (13/51, 25%) or flat (10/51, 20%), overall measuring a mean lesion size of 6.1 mm (range, 1.9–16 mm). Tumors occurred mostly on the lower eyelid (20/51, 39%) and medial canthus (14/51, 27%), with a low percentage found on the superior eyelid (9/51, 18%) and lateral canthus (8/51, 16%) (Table 1).

### 3.2. LC-OCT Features of Malignant Lesions of the Eyelid

Malignant Lesions Included 24 BCCs, 4 SCCs, 2 CMs

**Figure 1:**
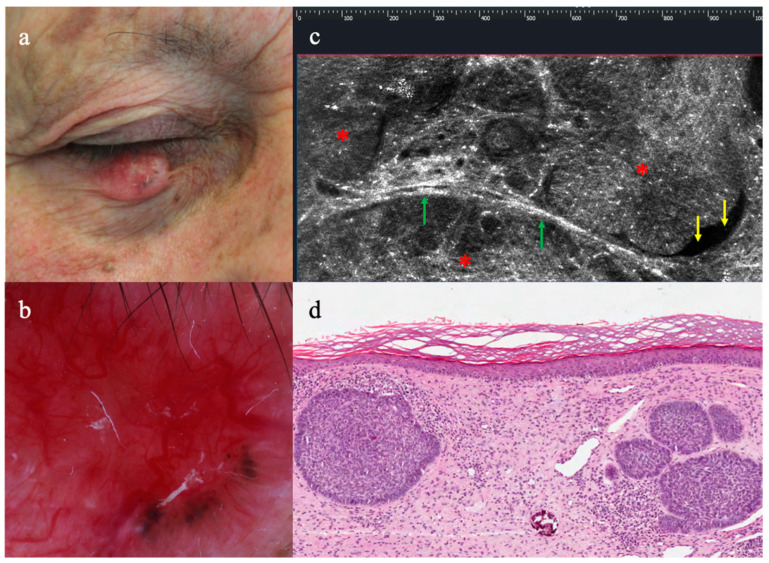
Clinical, dermoscopy, LC-OCT, and histopathology of nodular basal cell carcinoma on the lower eyelid of the right eye: (**a**) translucent nodule with visible telangiectasias, (**b**) revealing arborizing vessels and blue-grey ovoid nests in dermoscopy; (**c**) horizontal LC-OCT detecting bright islands of elongated tumour cells with polarized nuclei forming the typical peripheral palisade (red asterisks), surrounded by dark cleft-like spaces (yellow arrows) and highly refractile collagen bundles (green arrows), alike to classical histopathology (**d**).

**Figure 2:**
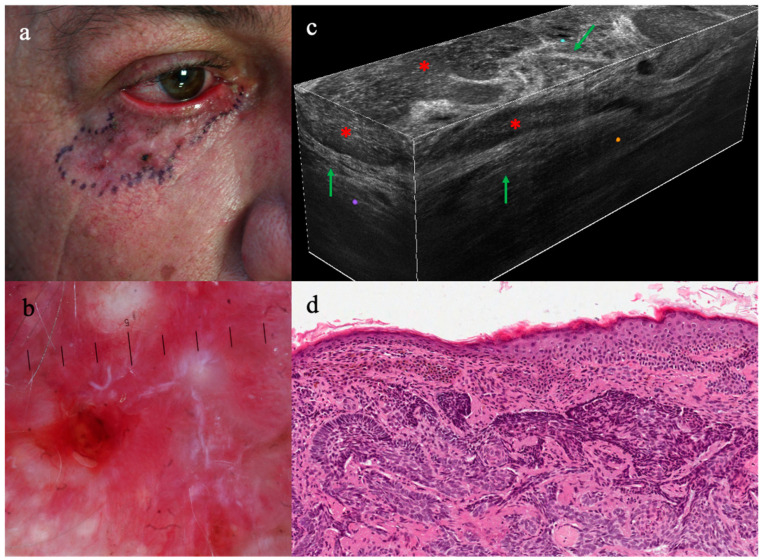
Clinical, dermoscopy, LC-OCT, and histopathology of infiltrative basal cell carcinoma on the lower eyelid and lateral canthus of the right eye: (**a**) ill-defined scar-like area with focal pigmentation, (**b**) exhibiting erosions, fine telangiectasis, and red and white structureless areas on dermoscopy; (**c**) LC-OCT 3D cube taken within the area ink-marked in Figure 1a, revealing dark elongated silhouettes (red asterisks), outlined by bright coarse collagen (green arrows) as exactly corresponding to the histopathologic image (**d**).

BCCs were clearly recognized for the presence of (i) dermal lobules (24/24, 100%) corresponding to tumor islands and consisting of a grey core defined as a hyper/hypo-reflective well-defined laminated structure with different shapes, featuring the so-called “millefeuille pattern”, dark rim, and peripheral bright rim, related to compression/alteration of the stroma outlining the tumor islands (Figure 1 and Figure 2). Additionally, (ii) enlarged peritumoral vessels (16/24, 66.6%), as well as epidermal changes, like (iii) thickening of the epidermis (6/24, 25%) and (iv) atypical keratinocytes (16/24, 14/24, 66%), have been observed according to BCC subtype.

**Figure 3:**
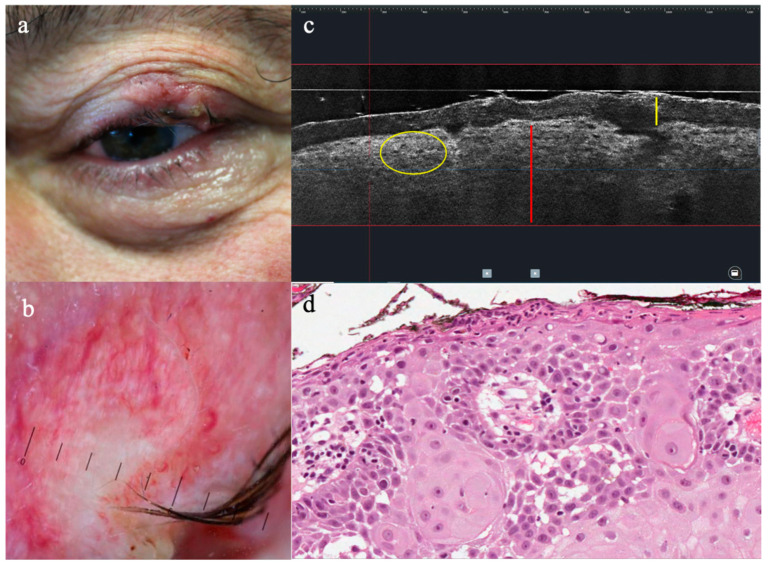
Clinical, dermoscopy, LC-OCT, and histopathology of squamous cell carcinoma on the upper eyelid of the left eye: (**a**) keratotic nodule with blood-spots and erosions; (**b**) dermoscopic features of ulceration, white structureless area and unfocused linear curved vessels; (**c**) vertical LC-OCT showing hyperkeratosis (yellow line), acanthosis hampering the visualization of the DEJ (red line) and epidermal pleomorphism of keratinocytes (yellow circle); (**d**) main histopathologic criteria of the tumour.

SCC was identified in LC-OCT for the presence of architectural alterations, as follows: (i) disarranged epidermis with atypical keratinocytes (4/4, 100%), (ii) hyperkeratosis (3/4, 75%) (iii) acanthosis (3/4, 75%) hampering the visualization of DEJ in invasive SCC, (iv) dilated vessels in the upper dermis (1/4, 25%) (Figure 3). Interestingly, two cases of hypertrophic AKs were diagnosed as SCC in LC-OCT, probably due to the overlapping features of these two entities, like the thickening of the epidermis with cellular pleomorphism, and not-visible DEJ.

In eyelid CMs, LC-OCT revealed (i) an irregular honeycomb pattern; and (ii) large, bright, roundish, and dendritic atypical cells along the basal layer and upward in the epidermis, unevenly distributed (2/2, 100%).

### 3.3. LC-OCT Features of Benign Lesions of the Eyelid

Benign lesions included nine SK, five scars, three hidrocystomas, two AK, one blue nevus, and one CH.

In SK, the main findings observed were (i) hyperkeratosis (8/9, 89%); (ii) acanthosis (8/9, 89%); (iii) bright roundish intraepidermal structures (pseudo-horn cysts) (7/9, 78%); and (iv) bright elongated/tubular structures (cords) (6/9, 67%). Scars were delineated by a network of bright, elongated structures that appeared thick and irregularly disposed in the whole dermis, relating to the poorly structured and densely packed collagen fibers, with no additional structures indicating a benign or malignant growth. One lesion was mistaken as BCC in LC-OCT.

**Figure 4:**
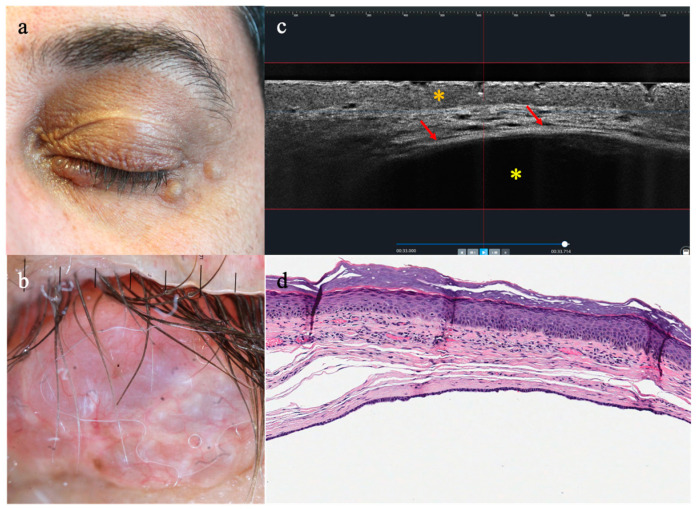
Clinical, dermoscopy, LC-OCT, and histopathology of apocrine hidrocystoma on the lower eyelid of the left eye: (**a**) translucent, dome-shaped nodule with a smooth surface; (**b**) dermoscopic findings of blue structureless areas and arborizing vessels; (**c**) vertical LC-OCT revealing a thin epidermis (orange asterisk) compressed below by a large oval dark area (yellow asterisk) lined by bright thick contour (red arrows), (**d**) closely resembling cystic space delineated by a double layer of epithelial cells in histopathology.

Hidrocystomas were all characterized in LC-OCT by the presence of (i) dark large areas expanding in the upper dermis, outlined by (ii) bright contours (3/3, 100%) (Figure 4). In AK were observed (i) pleomorphism of keratinocytes in the epidermis associated with (ii) a well-defined DEJ and (iii) dilated prominent vessels. Two cases resulted in hypertrophic AK in histopathology while being misjudged as invasive SCC in imaging. Blue nevus was misdiagnosed as an atypical melanocytic lesion, as shown in LC-OCT (i) dermal melanocytes with the classical sheet aspect, as well as (ii) plump bright cells scattered between collagen bundles. CH was characterized by a definite architecture with (i) lobular arrangement of dark round/oval areas outlined by (ii) fibrous septa.

### 3.4. Diagnostic Concordance LC-OCT/Histology

Diagnostic concordance between imaging and histopathology was 92.1% (47/51). Out of four cases of misdiagnoses with LC-OCT, two cases had a final diagnosis of hypertrophic AKs, one case of blue nevus, and one case of a surgical scar, being instead considered in LC-OCT 2 to beinvasive SCC, an atypical melanocytic lesion, and a BCC, respectively.

## 4. Discussion

In this retrospective study, we included a consecutive series of equivocal eyelid skin lesions, investigated by means of LC-OCT and surgically removed for histopathological examination, evaluating LC-OCT’s potential diagnostic role when compared to histology. Remarkably, LC-OCT allowed us to recognize the characteristic morphological findings of various benign and malignant lesions with an excellent concordance to histology.

The features we observed in malignant (BCC, SCC, CM) and some benign (SK, scar, AK, CH) lesions in this special area were in line with data reported in the literature in other cutaneous areas [17,19,22,23,24]

Indeed, as regards malignant lesions, BCC revealed its main characteristic findings, including dermal lobules defined by three components (“millefeuilles” pattern, middle dark rim, outer bright rim), peri-lobular vessels, and epidermal changes. These findings are strongly indicative of a diagnosis of BCC [17,24]. In the eyelid, in SCC, we observed epidermal changes (hyperkeratosis, acanthosis) with atypical keratinocytes, tumor extension downward in the dermis, and dilated vessels. In LC-OCT, the main differential diagnosis of SCC is represented by AK, which is identifiable by the presence of atypical keratinocytes confined in the epidermis, with a well-defined DEJ [23]. In CM, in LC-OCT, we detected atypical melanocytic cells, roundish and dendritic in shape, along the basal layer and upward in the epidermis with a pagetoid fashion, which accords with a recently reported single case of lentigo maligna of the face, detecting microscopic features in epidermis and around the hair follicles, as typically seen in LM [27].

For the benign lesions, eyelid SK showed architectural changes limited to the epidermis as follows: hyperkeratosis, acanthosis, pseudo-horn cysts, and hyperplasia of the basal layer, supporting the preliminary data of Lenoir et al. that first described the LC-OCT morphological features of a consecutive series of SK; in particular, in four subtypes of SK (flat, acanthotic, hyperkeratotic, and pigmented reticulated), the authors observed different LC-OCT architectural patterns, which appeared closely associated with the correspondent histological SK subtype [17]. In our series, scars were identified due to thickened collagen strands in the dermis with the absence of additional criteria indicating neoplastic growth. Often, in clinical settings, the accurate diagnosis of a scar is a challenge, as this lesion may resemble different cutaneous entities such as infiltrative basal cell carcinoma. In a recent study, the use of LC-OCT in a series of scar-like lesions allowed the authors to rule out a diagnosis of infiltrative basal cell carcinoma for the absence of typical BCC lobules, increasing the diagnostic performance for this type of entity [28]. Finally, in our study, CH was easily recognized thanks to the observation of a lobular pattern made of vascular lacunae expanding in the dermis. A series of benign and malignant skin vascular lesions have been recently investigated through the use of LC-OCT, resulting in this device being proven useful for the in vivo detection of an increased dermal vascularity with different morphologies in the various entities. Indeed, the correlation of LC-OCT patterns with histological substrates may provide practical clues for the identification of the vascular nature of a lesion and its differential diagnosis [18].

To our knowledge, this is the first description of hidrocystoma and blue nevus with LC-OCT, as the morphological findings of these entities have never previously been reported. In our cases of hidrocystomas, we observed well-outlined dermal cysts containing dark fluid material, compressing the above-thinned epithelium. The single case of blue nevus showed sheet dermal melanocytes, melanophages, and fibrosis. Future studies including a large series of blue nevi and hidrocystomas are warranted in order to support these preliminary observations.

Among the established non-invasive optical technologies that have gained popularity among both patients and physicians, dermoscopy is the most diffuse technique able to improve clinical examination, joined in recent years by handheld RCM, considered a reliable tool for in vivo diagnosis of the eyelid margin, as well as OCT for the identification of skin tumors in this area [7,11,12,13,14]. The chance offered by the new LC-OCT to acquire, in real time, both vertical and horizontal images, as well as 3D reconstruction, seems ideal for the investigation of this anatomical region, as this device can perform a global exploration of the lesion up to the superficial/mid-dermis [17,29]. The presence of an integrated dermoscopic camera that indicates the exact location of the probe while examining the lesion allows for a detailed assessment of tumor margins which, in some entities, do not correspond to clinical and dermoscopic extension (i.e., infiltrative BCC) [29]. In addition, LC-OCT may be beneficial in the assessment of disease recurrences after conservative or invasive treatment, being able to recognize microscopic structures indicative of a tumor [30]. Indeed, with this technique being non-invasive, easy to perform, painless, quick, and not requiring additional samples, it can be repeated several times during a follow-up schedule.

In this study, LC-OCT/histopathology concordance for diagnosis was as high as 92.1%, reaching high standards of diagnostic performance. By providing a sort of “virtual biopsy”, LC-OCT was able to visualize “in vivo” the main features of skin lesions closely resembling a classical histopathologic section, identifying different structures at the epidermis, DEJ, and dermis. Increasing studies in the literature report that a high LCT-OCT/histopathology correspondence of morphological substrates for both epithelial carcinomas (mainly BCC and SCC) and melanocytic lesions improves the meaning and interpretation of LC-OCT features in relation to skin tumours, hence reinforcing the utility of this new diagnostic tool [17,18,19,20,21,22,23,24,25,26,27,28].

The identification of key morphological criteria may provide advantages for an accurate distinction between malignant and benign lesions arising on the eyelids, impacting the management approach. It could be of noteworthy importance in a such special body site, as it is the eyelid area that is commonly considered to be where multimodal approaches originating in different medical specialties are required. Indeed, suspicious eyelid cancers are routinely discussed in multidisciplinary tumor meetings, with the involvement of physicians enrolled in different medical specialties (dermatologist, ophthalmologist, medical oncologist, radiation oncologist, pathologist, maxillofacial surgeons, and radiologists) gathering different treatment modalities (surgery, and/or radiotherapy, and/or systemic therapy) [10]. A non-invasive diagnosis influences the planning of the treatment, and, in the case of surgical procedures, its timing may give precise indications for a better definition of tumor margins. Moreover, further diagnostic work-up, like diagnostic biopsy or surgical excision with histopathological assessment, may be avoided.

The first limitation of this study was the sample size of skin lesions assessed with this new imaging diagnostic technique; however, skin lesions arising on the eyelids are quite rare, and the LC-OCT device, despite the rapidly expanding market, is currently available only in a few referral centers in Europe. In addition, as concerns technical issues, currently, the main limit for LC-OCT is depth acquisition (up to 500µm), potentially missing significative structures located deeper in the lesion. Such a limit may justify the misdiagnosis of two cases of AKs, considered invasive SCC in LC-OCT, due to the thickening of the epidermis not allowing the visualization of DEJ, and a case of blue nevus, whose features located in the dermis were misinterpreted as atypical nevus.

## 5. Conclusions

In conclusion, herein, we provide evidence supporting the use of LC-OCT for the evaluation of eyelid skin lesions. By integrating this new non-invasive imaging technique into the routine assessment of suspicious tumors of this area, LC-OCT imaging can increase clinical accuracy, further improving strategies adopted in multidisciplinary meetings and enhancing patient-centered care.

## Figures and Tables

**Table 1:** Clinical features of sample population and histopathological diagnosis.

	N (%)
Sex	
Male	23 (45%)
Female	28 (55%)
Mean age	66.4 (34–88)
Phototype	
I/II	32 (63%)
III	19 (37%)
Anatomical location	
Lower eyelid	20 (39%)
Medial canthus	14 (27%)
Upper eyelid	9 (18%)
Lateral canthus	8 (16%)
Clinical I	
Flat	10 (20%)
Slightly elevated	13 (25%)
Nodular	28 (55%)
Histopathology	
Malignant tumours	30/51 (59%)
BCC	24 (47%)
SCC	4 (8%)
Melanoma	2 (4%)
Benign tumours	21/51 (41%)
SK	9 (17%)
Scar	5 (10%)
Hidrocystoma	3 (6%)
AK	2 (4%)
Blue nevus	1 (2%)
Cherry hemangioma	1 (2%)

## Data Availability

The data presented in this study are available on request from the corresponding author.
